# Retrospective comparative clinical study on clinical effect of suture micromarsupialization on ranula

**DOI:** 10.2340/aos.v83.40492

**Published:** 2024-05-03

**Authors:** Bei-Bei Huo

**Affiliations:** Department of Dental, Beijing Zhongguancun Hospital, Beijing, China

**Keywords:** Sublingual gland cyst, micromarsupialisation, suture, sublingual gland resection

## Abstract

**Objectives:**

This study aimed to evaluate the clinical effect of suture micromarsupialisation on ranula.

**Methods:**

This is a retrospective comparative clinical study, the clinical data of 106 patients with simple ranula admitted to the Oral and Maxillofacial Surgery Department of Beijing Zhongguancun Hospital between August 2022 and September 30, 2023 were collected. The patients were divided into the research group (55 patients), who underwent suture micromarsupialisation, and control group (51 patients), who underwent ranula resections. The therapeutic methods were compared regarding cure rate, surgical duration, intraoperative blood loss, 24-h postoperative pain score, intraoperative and postoperative complications, and recurrence rate.

**Results:**

The difference in the total effective rate between the two groups was not statistically significant (98.18% vs. 96.08%, χ^2^ = 2.116, *p* = 0.347). Intraoperative blood loss (4.35 ± 1.19 vs. 26.33 ± 3.19), surgery duration (6.33 ± 1.43 vs. 26.33 ± 3.19) and the postoperative visual analogue scale score (0.32 ± 0.03 vs. 3.81 ± 0.15) in the research group were lower than in the control group (*p* *<* 0.05). The incidence rate of complications in the research group was lower than in the control group (7.27% vs. 25.49%, χ^2^ = 6.522, *p* = 0.011). The difference in the postoperative recurrence rate between the two groups was not statistically significant (3.63% vs. 9.80%, χ^2^ = 1.632, *p* = 0.201).

**Conclusions:**

Suture micromarsupialisation is a conservative therapeutic method for intraoral ranula. The cure rate of suture micromarsupialisation is similar to that of traditional surgery. It is recommended to use this technique as a first-line conservative therapeutic method for intraoral ranula, as it has the advantages of minimal invasion, simple operation, no pain, no need for haemostasis and no complications.

## Introduction

A sublingual gland cyst (SGC) is a form of salivary gland cyst that occurs in the sublingual gland. The exosmotic cyst is a type of pseudocyst without epithelial lining formed by acinus/duct rupture and mucus extravasation, accounting for approximately 98% of SGCs [1, 2]. Cysts are clinically divided into intraoral cysts, extraoral (latent) cysts and mixed (dumbbell-shaped) cysts according to their location. The intraoral cyst is the most common, accounting for approximately 70% of SGCs [3]. Clinical observation shows that the SGC consists of gland fluid that cannot be effectively removed due to sublingual gland duct blockage or salivary retention caused by local virus infection, foreign matter retention, trauma or salivary gland calculus, or purulent exudation caused by sublingual gland duct expansion/rupture or salivary extravasation [4]. An SGC usually occurs between the mucous glands on the ventrum of the tongue tip and the sublingual gland. The clinical symptoms include a light red lump under the skin mucosa at the bottom of the mouth. The lump has a thin cyst wall with a delicate texture. The increase in cyst volume can affect the submentum and even the bottom of the mouth, thus seriously hindering the normal speaking, breathing and eating of patients and reducing their quality of life [5].

Researchers have confirmed [6] that there are two therapeutic methods suitable for SGCs. The first is to surgically remove the cyst and drain the cyst fluid. The second method involves inducing local tissue fibrosis or inhibiting sublingual gland extravasation and blocking mucus extravasation to eliminate the cyst. Currently, sublingual gland resection and the radical treatment of SGCs are commonly used in clinical practice. However, surgery may lead to damage to the submandibular gland duct or lingual nerve and bleeding [7]. Marsupialisation was once considered a first-line method to eradicate SGCs; however, this method has a high rate of postoperative recurrence and a high risk of triggering iatrogenic latent SGCs [8, 9]. With the advancement of medical technology, improved marsupialisation or other new minimally invasive techniques are used for the treatment of SGCs, which not only reduces the postoperative recurrence rate but also achieves improved safety [10, 11]. However, there are some shortcomings in the therapeutic effect and postoperative recovery. In this study, based on the clinical data of all patients with SGCs treated at Beijing Zhongguancun hospital, the clinical effects of double suture marsupialisation and traditional sublingual cyst resection on SGCs were compared between two groups, thereby providing support for the scientific treatment of patients with SGCs.

## Patients and methods

### Patients

This is a retrospective comparative clinical study. The clinical data of 106 patients with simple SGCs admitted to the Oral and Maxillofacial Surgery Department of Beijing Zhongguancun Hospital (BZH) between August 2022 and September 30, 2023 were collected. The patients were divided into the research group (55 patients), who underwent suture micromarsupialisation, and the control group (51 patients), who underwent SGC resections. The diagnostic criteria were subject to the Guidelines for the Diagnosis and Treatment of Oral Diseases [12]. The inclusion criteria were as follows: patients diagnosed with SGCs through visual examination, palpation, puncture or imaging diagnosis; patients with intraoral SGCs that only involve unilateral sublingual glands; patients who voluntarily participated in this retrospective study and signed the informed consent form. The exclusion criteria were as follows: patients with lesions involving bilateral sublingual glands; patients with severe systemic diseases, such as active gastrointestinal, kidney and liver diseases, coagulation disorders or heart failure; patients with incomplete clinical data; patients who were lost to follow-up ; patients with mental illness or communication disorders; patients with combined organ dysfunction, haematological system diseases, immune system diseases or malignant tumours; pregnant and lactating female patients. Ethical approval was obtained from the Ethics Committee of BZH, with the ethical code of NXXXX1. All the patients included in the present research signed the written consent form.

### Choice of treatment method for ranula

The choice of surgical treatment method for an SGC (also known as a submandibular gland cyst) depends on the size, location and specific characteristics of the cyst, as well as the patient’s overall health. The following is a selection of surgical treatment options that may be considered. (1) Cystectomy: This involves the complete removal of the cyst and, in some cases, the affected portion of the sublingual gland. This is a common approach for larger cysts or those that have caused significant symptoms. The surgery can be performed through a small incision in the mouth or externally on the neck, depending on the cyst’s location and size. (2) Marsupialisation: This is a surgical technique where the cyst is partially removed and the remaining cyst lining is sutured to the oral mucosa, creating a controlled opening that allows continuous drainage. This method may be suitable for certain types of cysts. (3) Sialoadenectomy: In some cases, when the sublingual gland is severely affected or when the cyst keeps recurring, a complete removal of the sublingual gland may be necessary. This is typically considered when other treatments have not been successful. (4) Endoscopic surgery: This involves using a thin, flexible tube with a camera and surgical instruments to access and remove the cyst through small incisions in the mouth. This approach is less invasive and may result in faster recovery. (5) Transoral or transcutaneous drainage: In some situations, the surgeon may choose to drain the cyst through a small incision or puncture using a fine needle or catheter to relieve symptoms temporarily or before performing definitive surgery.

### Methods

The SGC resection group was treated as follows. First, the patient was placed in a horizontal supine position and given general anaesthesia through tracheal intubation. An incision was made outside the sublingual fold. The mucosal tissue at the bottom of the mouth was cut upwards along the surface of the cyst using the intraoral approach, and the incision was maintained parallel to the mandibular lingual gingiva. The mucosa was gently stripped off, and the surrounding glands and cyst wall were removed. Focus was kept on the position and orientation of the lingual nerve and submandibular gland duct, the submandibular gland duct was protected and the sublingual gland and cyst were completely removed. The main duct of the sublingual gland was sheared off, the bleeding was stopped, the surgery field was cleaned with sterile physiological saline and the wound was sutured.

The research group was treated as follows. First, the patient was placed in a semi-supine position and given topical anaesthesia with 1% lidocaine hydrochloride. A thin rubber drainage strip was inserted into a round needle and an interrupted suture was conducted through the cystic cavity in a horizontal direction. A distance of 2 mm was maintained between the entry and exit points and the cyst, with a needle space of approximately 5 mm. Care was taken not to tie knots too tightly or to suture under tension to avoid the cyst wall breaking or the rubber drainage strip falling out prematurely. After suturing, an iatrogenic fistula was formed at the rubber drainage strip, allowing continuous drainage of the cyst fluid. After surgery, the patient used a compound chlorhexidine gargle for 1 week and then returned to the clinic for the follow-up visit. If the cyst was significantly alleviated and there was significant cyst fluid drained from the iatrogenic fistula, a good drainage effect was obtained. During the follow-up visit 2 weeks after surgery, if there was cyst regression and no cyst fluid drained from the iatrogenic fistula, the patient’s rubber drainage strip was removed. If there was cyst fluid drained from the iatrogenic fistula, the drainage strip was retained for 1 week. If there was no complete cyst regression after 4 weeks, a thicker rubber strip was used for suturing. If there was still no complete cyst regression after 4 weeks of observation, the affected sublingual gland and cyst were removed under general anaesthesia. The above operations were all completed by the surgeons in the medical group.

### Data collection

The following baseline data were collected from the patients: sex, age, height, smoking history, drinking history, body mass index (BMI), hypertension history and diabetes history. The clinical efficacy indexes were as follows: cured – the SGC disappeared, the mucosa at the bottom of the mouth was normal and there was no obvious limitation or discomfort during tongue activity, but the tongue had a hard texture; effective – the SGC was reduced by 60%–90%, and tongue activity was significantly improved; noneffective – the SGC was reduced by < 59%, there was no significant improvement in tongue activity and it was necessary to conduct observation or surgery again; total effective rate = (number of cured cases + number of effective cases) / total number of cases × 100%. The surgery-related indexes were as follows: intraoperative blood loss, surgery duration and 24-h postoperative pain score; the visual analogue scale (VAS) was used to evaluate postoperative pain response, with each patient giving a score of 0–10 based on increasing degrees of pain. The following complications during and after surgery were recorded: bleeding, numbness at the tongue tip, discomfort at the bottom of the mouth and duct damage or obstruction. The recurrence-monitoring procedures were as follows: follow-up visits on all patients were conducted for 2 months, and any recurrence was recorded; recurrence rate = number of recurrent cases / total number of cases × 100%. The recurrence criteria were as follows: after 2 months, the cyst did not completely disappear, or the cyst reappeared.

### Statistical analysis

The SPSS v. 25.0 statistical software package was used for data processing. The metrological data of normal distribution were expressed as x ± s. The *t*-test was conducted for analysis, and the counting data were represented by frequency (*n*) and percentage (%). The χ^2^ test was conducted. The statistical significance levels of all statistical tests were defined as *p* < 0.05.

## Results

### General data

The comparison results of baseline data between the two groups showed that there was no statistically significant difference in sex, age, smoking history, cyst diameter, drinking history, BMI, hypertension history or diabetes history (*p* > 0.05). The two groups of patients were comparable, as shown in Table 1.

**Table 1:** Comparison of baseline data between the two groups.

Clinical data	The research group (*n* = 55)	The control group (*n* = 51)	χ^2^/t	*P*
Gender (male/female)	25/30	21/30	0.197	0.657
Age (years old, x ± s)	26.33 ± 3.19	27.12 ± 4.36	1.371	0.172
Height (cm, x ± s)	159.32 ± 8.89	160.81 ± 11.94	1.122	0.305
BMI (kg/m^2^, x ± s)	19.52 ± 7.63	20.32 ± 8.75	0.798	0.440
Cyst diameter	2.25 ± 0.36	2.46 ± 0.23	0.562	0.578
Smoking history (number of cases)	21	18	0.095	0.758
Drinking history (number of cases)	17	15	0.028	0.867
Hypertension history (number of cases)	6	4	0.291	0.589
Diabetes history (number of cases)	7	5	0.225	0.635

### Comparison of clinical effects between the two groups

The comparison results of the clinical effects between the two groups showed that in the research group, 48 patients were cured, six patients were effective and one patient was ineffective; the total effective rate was 98.18%. In the control group, 39 patients were cured, 10 patients were effective and two patients were ineffective; the total effective rate was 96.08%. The difference between the two groups was not statistically significant, as shown in Table 2.

**Table 2:** Comparison of clinical effects between the two groups.

Group	*n*	Cured	Effective	Non effective	Total effective rate
The research group	55	48	6	1	98.18%
The control group	51	39	10	2	96.08%
χ^2^					2.116
*P*					0.347

### Comparison of surgery-related indexes between the two groups

The comparison results of surgery-related indexes between the two groups showed that intraoperative blood loss was 4.35 ± 1.19 mL, surgery duration was 6.33 ± 1.43 min and the postoperative VAS score was 0.32 ± 0.03 in the research group. In the control group, intraoperative blood loss was 26.33 ± 3.19 mL, surgery duration was 47.12 ± 6.36 min and the postoperative VAS score was 3.81 ± 0.15. The difference between the two groups was statistically significant (*p* < 0.001), as shown in Table 3.

**Table 3:** Comparison of surgical related indexes between the two groups.

Clinical data	The research group (*n* = 55)	The control group (*n* = 51)	*t*	*P*
Intraoperative blood loss(mL)	4.35 ± 1.19	26.33 ± 3.19	28.111	< 0.001
Surgery duration (min)	6.33 ± 1.43	47.12 ± 6.36	26.568	< 0.001
Postoperative VAS score	0.32 ± 0.03	3.81 ± 0.15	33.510	< 0.001

VAS: visual analogue scale.

### Comparison of postoperative complications between the two groups

The comparison results of the postoperative complications between the two groups showed that there was one patient with haematoma and bleeding, one patient with a postoperative infection and two patients with tongue numbness in the research group; the incidence rate of complications was 7.27%. In the control group, there were four patients with haematoma and bleeding, three patients with submandibular gland duct injury, two patients with postoperative infection and four patients with tongue numbness; the incidence rate of complications was 25.49%. The difference between the two groups was statistically significant, as shown in Table 4.

**Table 4:** Comparison of postoperative complications between the two groups.

Clinical data	The research group (*n* = 55)	The control group (*n* = 51)	χ^2^	*P*
Total number of complications	4	13	6.522	0.011
Hematoma and bleeding	1	4	-	-
Submandibular gland duct injury	0	3	-	-
Postoperative infection	1	2	-	-
Tongue numbness	2	4	-	-

### Comparison of postoperative recurrence rate between the two groups

Six months later, follow-up visits were made to the two groups of patients to obtain recurrence rates. The results showed that recurrence was observed in two patients in the research group, showing a recurrence rate of 3.63%. In the control group, recurrence was observed in five patients, showing a recurrence rate of 9.80%. The difference in the postoperative recurrence rate between the two groups was not statistically significant (χ^2^ = 1.632, *p* > 0.05), as shown in Table 5.

**Table 5:** Comparison of recurrence rate between the two groups.

Group	*n*	Recurrence	No recurrence	Recurrence rate	χ^2^	P
The research group (*n* = 55)	55	2	53	3.63%	1.632	0.201
The control group (*n* = 51)	51	5	46	9.80%		

## Discussion

Ranula are usually pseudocysts without epithelial cell lining caused by the rupture of small secretion ducts in the sublingual gland acinus and a large amount of mucus extravasation. The therapeutic methods for SGCs include routine surgery, sclerotherapy, laser therapy, oral medication and non-surgical therapy [13, 14]. Sublingual gland resection is commonly used in clinical practice; however, its surgical field is small and the surrounding anatomical structure is complex, which may lead to complications such as lingual nerve injury and submandibular gland duct injury [8].

Some experts have suggested that sublingual gland resection is only suitable for recurrent patients, and conservative surgery is feasible in the initial diagnosis of SGCs [15–17]. Harrison et al. [6] noted that small ducts with mucus extravasation can be blocked in the conservative treatment of SGCs [18]. Another method is to drain the cyst fluid. Therefore, the main task is to propose efficient, minimally invasive, non-invasive and safe non-surgical therapeutic methods.

Marsupialisation was once used for the treatment of SGCs and was considered a first-line therapeutic method; however, traditional marsupialisation had a high postoperative recurrence rate (approximately 16.7%–66.7%) [3, 19], and there was a risk of developing iatrogenic extraoral SGCs. Morton et al. [20] proposed the method of suturing the top of the cyst with silk thread and cured four patients with SGCs. Delbem et al. [21] named this therapeutic method micromarsupialisation. Piazzetta et al. used this therapeutic method in the treatment of oral mucinous gland cysts [22]. The effective rate was >85%, and the recurrence rate of this therapeutic method was not significantly different from that of traditional surgical resection. The principle of suture micromarsupialisation is to use the suture to pass through the cyst and create a sinus near the suture so that the viscous cyst fluid in the cyst drains out along the sinus and granulation tissue gradually forms on the cyst wall, which is a gradual healing process [23]. Furthermore, saliva formed by sublingual glands is cleaned after drainage through the sinus tract and is reformed after blocking the sinus.

The experimental results showed that preoperative blood loss, surgery duration and the postoperative VAS score between the two groups were statistically significant, and the indexes of the research group were significantly lower than those of the control group. Moreover, the postoperative effect indexes of the research group were lower than those of the control group. The results indicated that in the treatment of SGCs, as well as ensuring excellent efficiency, micromarsupialisation can improve surgery-related indexes, relieve the pain of patients and reduce complications. The reason is that suture micromarsupialisation is usually performed on thin cyst walls and swollen surfaces, and the operation process is simple, requiring only the injection of anaesthesia gas into the surface cavity [24], thereby improving the sensory effect of patients. During SGC resection, the surrounding tissues are difficult to remove due to the thin cyst walls, and the surgical field has a small volume. Therefore, clamping and suturing cause the narrowing of the submandibular gland duct, and incorrect shearing may damage the submandibular gland duct [25]. In addition, the bottom of the mouth has a complex anatomical structure. If the hypoglossal blood vessels or nerves are not accurately identified during surgery, perioperative haematoma or bleeding may occur, as well as damage to the hypoglossal nerve and postoperative tongue numbness [8].

However, the present study has certain limitations. First, this single-centre study may not maintain baseline consistency during the comparison between two groups, and the patients may have other comorbidities that can affect prognosis. Second, the small sample size included in the present study may lead to insufficient testing effectiveness. In subsequent research, the sample size should be increased. Third, the follow-up management varied for different patients, and if the impact is eliminated, deviation in the results may occur. Finally, in retrospective research, it is not easy to distinguish chronological order, which may reduce the credibility of the results. In the future, multi-centre prospective studies with a large sample size should be conducted.

In conclusion, as a conservative therapeutic method for intraoral SGCs, the cure rate of double suture micromarsupialisation is similar to that of traditional surgery. Since it has the advantages of minimal invasion, simple operation, no pain, no need for haemostasis and no complications, it is recommended as a first-line conservative therapeutic method for intraoral SGCs

## Declarations

### Availability of data and materials

The datasets used and analyzed during the current study are available from the corresponding author on reasonable request.
